# DDI‐Transform: A neural network for predicting drug‐drug interaction events

**DOI:** 10.1002/qub2.44

**Published:** 2024-04-29

**Authors:** Jiaming Su, Ying Qian

**Affiliations:** ^1^ School of Computer Science and Technology Shanghai Frontiers Science Center of Molecule Intelligent Syntheses East China Normal University Shanghai China

**Keywords:** adaptive learning, graph convolutional networks, interaction prediction, meta‐path

## Abstract

Drug‐drug interaction (DDI) event prediction is a challenging problem, and accurate prediction of DDI events is critical to patient health and new drug development. Recently, many machine learning‐based techniques have been proposed for predicting DDI events. However, most of the existing methods do not effectively integrate the multidimensional features of drugs and provide poor mitigation of noise to get effective feature information. To address these limitations, we propose a DDI‐Transform neural network framework for DDI event prediction. In DDI‐Transform, we design a drug structure information feature extraction module and a drug bind‐protein feature extraction module to obtain multidimensional feature information. A stack of DDI‐Transform layers in the DDI‐Transform network module are then used for adaptive learning, thus adaptively selecting the effective feature information for prediction. The results show that DDI‐Transform can accurately predict DDI events and outperform the state‐of‐the‐art models. Results on different scale datasets confirm the robustness of the method.

## 1 INTRODUCTION

With the rapid growth in the number of drug classes, it is critical to manage drug safety when multiple drugs will be used in the treatment of a single disease. When two or more drugs are taken together, they can trigger unexpected side effects, adverse reactions, and even serious toxicity [1]. The pharmacological effects triggered by two or more drugs taken together in treatment are called drug‐drug interactions (DDIs). Such interactions can have clinical manifestations that have the potential to increase or decrease the therapeutic effects of the drugs [2, 3]. Therefore, in order to prevent unexpected DDI events, effective identification of potential DDIs is crucial, especially during the development of new drugs, and is important for both patients and the pharmaceutical industry.

However, the detection of DDI for multiple drug pairs in the assay remains both time consuming and expensive [4]. In the technological context of computer‐aided drug research, an increasing number of computational methods are being successfully used for DDI prediction [5]. In the last decade, the accumulation of experimentally determined DDI entries has facilitated the application of computational methods to find potential DDIs [6], especially machine learning based methods. Various machine learning methods have proven to offer promising approaches for providing preliminary screening of DDI for further experimental validation, with the advantages of high efficiency and low cost. Some approaches extract different kinds of drug features and then use a series of similarity measures to predict DDI [7, 8, 9]. Examples of such approaches include implementation of multi‐task learning on DDI type prediction [10, 11], multi‐type DDI pharmacological action prediction using knowledge graph summarization [12], as well as some efforts to predict DDI using multiple data sources [5, 13, 14]. The research efforts aimed at improving DDI prediction can take two directions: integration of multiple drug features and application of deep learning techniques.

Many methods calculate similarities of drug features by integrating multiple data sources and perform prediction of DDI by fusing similarities, based on the hypothesis that similar drugs tend to interact with each other [7, 15, 16]. These methods extract information about different kinds of drug features, such as chemical structure information, physical property information and phenotypic property information, and then use similarity measures. Gottlieb et al. [17] used different types of drug‐drug similarity (chemical and side effect similarity) to perform statistical validation. Vilar et al. [18] developed a new method for predicting DDI based on the similarity of molecular structural properties and it can be applied to large‐scale datasets. Deng et al. [5] proposed a DDIMDL framework to combine various drug features in a model to predict DDI events. Takeda et al. [3] predicted DDI by an interaction network consisting of pharmacodynamic and pharmacokinetic properties and based on structural similarity. Lin et al. [19] proposed an Multi‐type Drug‐Drug Interactions via Supervised Contrastive Learning framework to combine various drug features to execute supervised contrastive learning.

Deep learning models require a large amount of data for training, and effective features are extracted to predict DDI by using SMILES sequences, molecular fingerprints, and molecular graphs as inputs to the model. Ryu et al. [20] proposed a deep learning framework to predict DDI by using drug molecular structures. Huang et al. [8] developed a dictionary learning framework using chemical substructure. Gómez Bombarelli et al. [21] proposed a variational auto‐encoder to encode SMILES sequences. Multi‐Label Robust Disentangling Autoencoders [22] effectively exploits unlabeled drug information and uses a multi‐task semi‐supervised learning framework.

Although some satisfactory progress has been made by existing methods, most of them do not thoroughly integrate the features of drugs in multiple dimensions and cannot accurately identify which information is valid for model prediction. Moreover, some methods that use graph neural networks directly on heterogeneous graphs are prone to involving noisy information, leading to biased predictions.

To address the above limitations, this work focuses on fusing drug multidimensional features and acquiring effective feature information through adaptive learning. We propose a DDI‐Transform neural network framework to perform DDI event prediction. In the DDI‐Transform neural network framework, we design a drug structure information feature extraction module and a drug bind‐protein feature extraction module to obtain drug multidimensional feature information. Then a stack of DDI‐Transform layers in the DDI‐Transform network module are used for adaptive learning, so as to learn a new subgraph and then perform graph convolutional network (GCN) operations to obtain drug features. The prediction is then performed in the interaction prediction module. The main contributions of our work are summarized as follows:

1. We design a DDI‐Transform network module to fuse drug multidimensional features, that is, drug structure feature and drug bind‐protein feature. The module is able to use adaptive learning to adaptively select the feature information that is valid for prediction.
2. Instead of using GCN operations directly on the interaction network, we using GCN operations on the subgraphs learned by adaptive learning can largely select the effect information and improve the prediction accuracy.
3. We conduct extensive experiments on two real‐world datasets to demonstrate the effectiveness of our model compared with classic and the state‐of‐the‐art (SOTA) methods.

## 2 RESULTS

**FIGURE 1:**
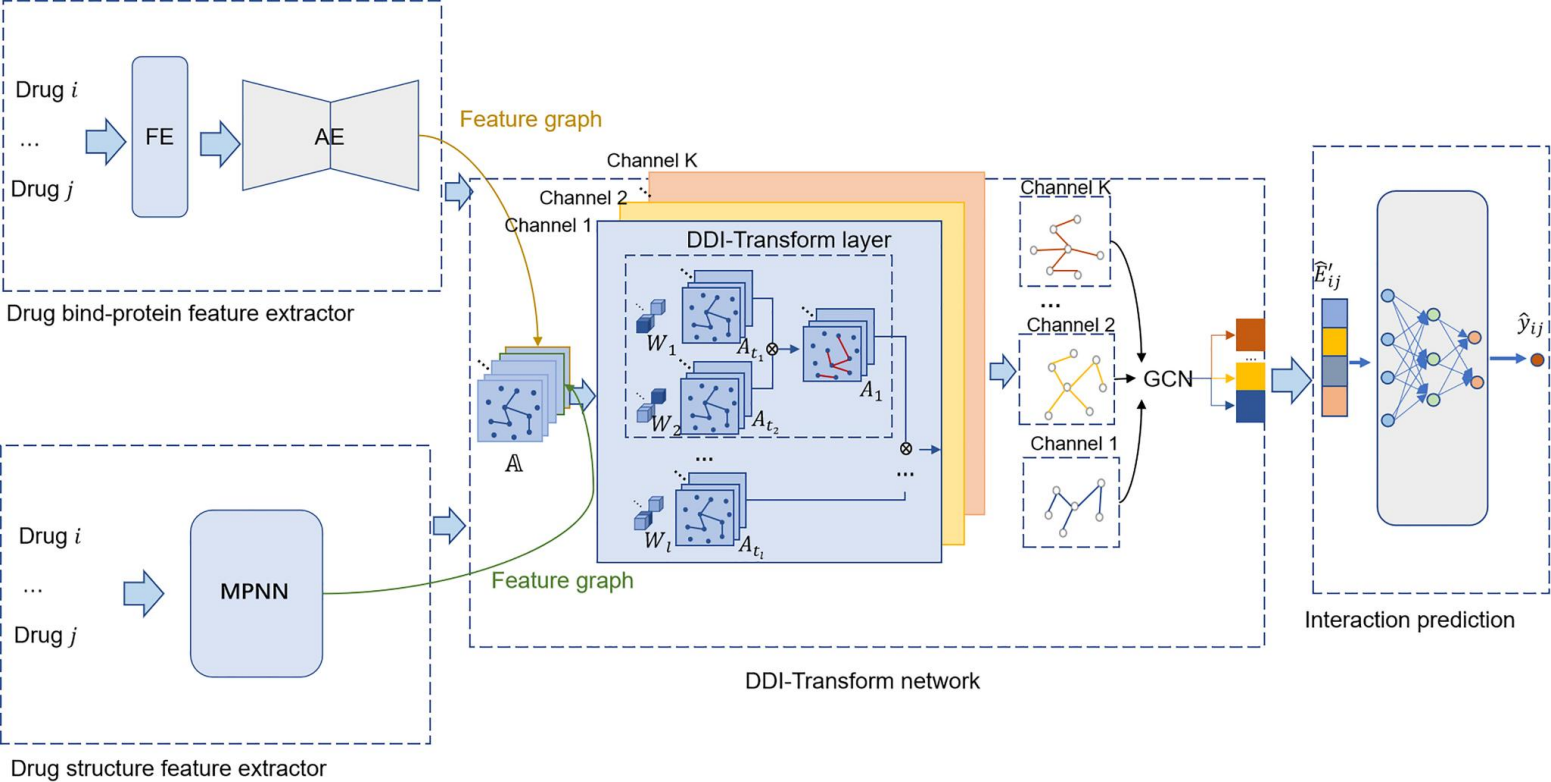
Overview of the proposed DDI‐Transform model, consisting of four blocks: drug bind‐protein feature extractor, drug structure feature extractor, DDI‐Transform network and interaction prediction. AE, auto‐encoder; DDI‐Transform network, drug‐drug interaction transform network; FE, feature extraction; MPNN, message passing neural network.

Figure 1 shows the overview of the DDI‐Transform framework. The drug structure feature extraction module utilizes the message passing network with an attention pooling mechanism to encode drug structure information. The drug bind‐protein feature extraction module uses information about the drug bind‐proteins (target, enzyme, etc.) associated with the drug, and uses similarity functions and autoencoders to encode the drug bind‐proteins to obtain the external features of the drug. The DDI‐Transform network module fuses the two types of features, and then the meta‐path‐inspired DDI‐Transform network performs adaptive learning to obtain the final drug features. The drug features are then fed into a multilayer perceptron (MLP) for DDI event prediction.

### 2.1 Comparative analysis

In this study, our model is compared with the following, including recent SOTA methods:

**MDF‐SA‐DDI** [13]: the MDF‐SA‐DDI uses multiple data sources fused into multiple models, including Siamese network, convolutional neural network, and autoencoder. The obtained drug multidimensional features are then fed into a transformer with a self‐attention mechanism for fusion learning and prediction.
**MDNN** [14]: Multimodal deep neural network (MDNN) mainly uses the heterogeneous features of drugs to build a drug knowledge graph, where drug node features are learned directly using graph neural networks and combined with heterogeneous features to predict DDI events.
**DDIMDL** [5]: DDIMDL uses multiple heterogeneous features of the drug and calculates the similarity between various features of the drug using a similarity function and feeds them into a deep neural network (DNN) for learning and predicting.
**DeepDDI** [20]: DeepDDI feeds structural information of drug pairs into a DNN for learning and predicting DDI events.
**Deep neural network** [23]: DNN in which drug features from a dataset are concatenated and fed into a DNN to learn and predict.
**Random forest (RF)** [24]: RF concatenates drug features from the dataset and feeds them into multiple decision trees for training and predicting.
**K‐nearest neighbor (KNN)** [25]: KNN is used to find the nearest K classes by calculating the distance of a new DDI record from other records; the classification of the record is determined by the majority of K classes thus predicting DDI events.
**Logistic regression (LR)** [26]: LR is used as a predictive probability by introducing a sigmoid function in a linear regression model that maps the output value between 0 and 1. This is similar to a single‐layer neural network.

In this paper, the proposed method is experimentally compared with other methods on two different scale datasets. Overall, the experimental results of the proposed method in this paper on two different scale datasets consistently outperform the traditional machine learning methods and the current more advanced deep learning methods. The following are the specific results of the analysis of the experiment.

Table 1 presents the experimental results of the proposed method and other comparative methods on Dataset 1. It shows that all current more advanced deep learning methods consistently outperform traditional methods in all metric values. The method proposed in this paper significantly outperforms traditional machine learning methods with at least 16.69%, 14.47%, 0.32%, and 32.25% improvement in Accuracy (ACC), Area Under the Precision‐Recall Curve (AUPRC), Area Under Curve (AUC), and F1 metrics, respectively. The method proposed in this paper has at least 2.88%, 1.10%, 0.03%, and 2.95% improvement in ACC, AUPRC, AUC, and F1 metrics compared to deep learning methods that are popular in current research. In particular, it should be noted that the F1 metric outperforms the existing deep learning methods because it can characterize the model performance in a more balanced way. The 2.95% performance improvement in the F1 metric shows the effectiveness of the proposed method.

**TABLE 1:** the performance on two datasets.

Dataset	Methods	ACC	AUPR	AUC	F1
Dataset 1	LR	0.7920	0.8400	0.9960	0.5948
	KNN	0.7214	0.7716	0.9813	0.4831
	RF	0.7775	0.8349	0.9956	0.5936
	DNN	0.8797	0.9134	0.9963	0.7223
	DeepDDI	0.8371	0.8899	0.9961	0.6848
	DDIMDL	0.8852	0.9208	0.9976	0.7585
	MDNN	0.9175	0.9668	0.9984	0.8301
	MDF‐SA‐DDI	0.9301	0.9737	0.9989	0.8878
	**DDI‐Transform**	**0.9589**	**0.9847**	**0.9992**	**0.9173**
Dataset 2	LR	0.5229	0.5288	0.9805	0.2373
	KNN	0.5797	0.5964	0.8998	0.3805
	RF	0.6956	0.7567	0.9892	0.5760
	DNN	0.7908	0.8539	0.9949	0.7649
	DeepDDI	0.7211	0.7724	0.9914	0.6854
	DDIMDL	0.9229	0.9637	0.9993	0.9105
	MDNN	0.9245	0.9706	0.9995	0.9112
	MDF‐SA‐DDI	0.9291	0.9773	0.9996	0.9117
	**DDI‐Transform**	**0.9435**	**0.9896**	**0.9997**	**0.9345**

*Note*: ACC: measures the proportion of correctly classified instances among all instances. AUPR: summarizes the trade‐off between precision and recall for different threshold values in binary classification. AUC: measures the ability of a model to distinguish between classes, plotting true positive rate against false positive rate. F1: harmonic mean of precision and recall, providing a balance between the two metrics in binary classification. And the bold values mean the best performance in all methods (Applicable to Table 2).

Abbreviations: DDIMDL, drug‐drug interaction multimodal deep learning; DeepDDI, deep drug‐drug interaction; DNN, deep neural network; KNN, K‐nearest neighbor; LR, logistic regression; MDF‐SA‐DDI, multi‐source drug fusion self‐attention drug‐drug interaction; MDNN, multimodal deep neural network; RF, random forest.

Table 1 also posts the experimental results on Dataset 2. Again, it shows that all current more advanced deep learning methods consistently outperform traditional methods in all metric values. The method proposed in this paper still consistently outperforms traditional machine learning methods and the best available deep learning methods on Dataset 2. There is at least 24.79%, 23.29%, 1.05%, and 35.87% performance improvement, respectively, in ACC, AUPRC, AUC, and F1 metrics compared to the traditional method. This paper’s method also provides at least 1.44%, 1.23%, 0.01%, and 2.28% improvement in ACC, AUPRC, AUC, and F1 metrics, respectively, over the popular deep learning methods. The effectiveness of the proposed method is further confirmed by the fact that the F1 value metric is 2.28% higher than that of the current model MDF‐SA‐DDI, which achieves SOTA performances. It can be observed that in Dataset 2, the F1 value index is 2.28% higher than the best model, compared to Dataset 1. The amount of data in Dataset 2 is larger and there are more types of drugs, which increases noisy data, thus reducing the model prediction effectiveness. The results of the method proposed in this paper outperform those of the existing methods on two different scale datasets, indicating that the proposed method in this paper has some robustness.

## 3 discussion

### 3.1 Ablation study

To explore how the DDI‐Transform model proposed in this paper improves the results and the role of specific components, we perform ablation study on the model, DDI‐Transform‐f and DDI‐Transform‐d. DDI‐Transform‐f keeps only the two feature extraction modules and the interaction prediction module, and removes the DDI‐Transform network module for training the model to predict DDI events. Meanwhile, the DDI‐Transform‐d removes the two feature extraction modules, but keeps the DDI‐Transform network module and the interaction prediction module for training the model.

Table 2 lists the experimental results, showing that our proposed model DDI‐Transform consistently outperforms DDI‐Transform‐f and DDI‐Transform‐d, which lack the adaptive learning and two feature extractors, respectively. In terms of specific quantification, for the DDI‐Transform over the DDI‐Transform‐f there is an 11.42% improvement in the F1 metric on Dataset 1 and an 8.29% improvement in the F1 metric on Dataset 2, indicating that the adaptive learning of the proposed method occupies a very important role. This may be the reason why DDI‐Transform outperforms other multiple data source fusion methods such as MDF‐SA‐DDI and MDNN. The difference in metric values further indicates that noisy information is still present on the large‐scale dataset. The difference in the F1 metric between DDI‐Transform‐d and DDI‐Transform‐f shows that the DDI‐Transform network module offers the main contribution to the improvement of the model effect. On the other hand, it also shows that the model is able to capture the effective association information between DDI events in an adaptive way, and the process is achieved by learning new meta‐path subgraphs to fuse the effective association information. The enhancements on two datasets of different scales also further confirm the robustness of the proposed method.

**TABLE 2:** the ablation study on Dataset 1 and Dataset 2.

Dataset	Algorithm	Performance			
		ACC	AUPR	AUC	F1
Dataset 1	DDI‐Transform‐f	0.8937	0.9281	0.9976	0.8031
	DDI‐Transform‐d	0.9396	0.9637	0.9981	0.8769
	DDI‐Transform	**0.9589**	**0.9847**	**0.9992**	**0.9173**
Dataset 2	DDI‐Transform‐f	0.8896	0.9032	0.9958	0.8516
	DDI‐Transform‐d	0.9265	0.9527	0.9971	0.9024
	DDI‐Transform	**0.9435**	**0.9896**	**0.9997**	**0.9345**

### 3.2 Parameter sensitivity analysis

**FIGURE 2:**
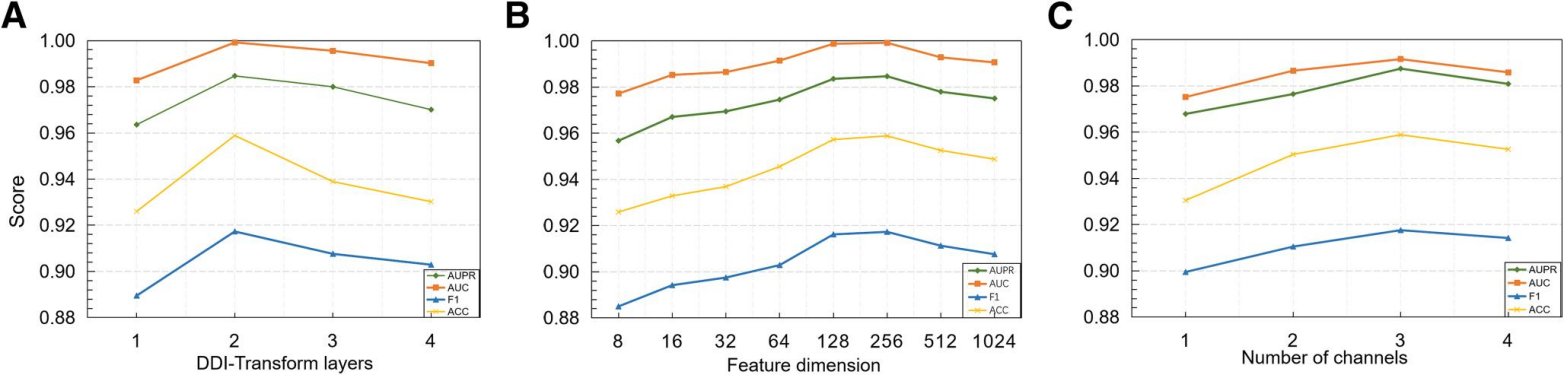
The metric scores under different hyper‐parameters.

The hyper‐parameters could influence the performances of DDI‐Transform. Therefore, we discuss three hyper‐parameters in Dataset 1: DDI‐Transform layers, number of channels and feature dimension. We fix other parameters when studying the effect of each of them. The experimental results are shown in Figure 2.

We vary the number of the DDI‐Transform layers to explore the effectiveness of the DDI‐Transform. From Figure 2A, we can see that our model achieves the best results when the number of DDI‐Transform layers is equal to 2. When the number of DDI‐Transform layers is too small, the model is not able to fully integrate the effective feature information. When the number of layers is too large, the model tends to incorporate the noise information.

We vary the size of the feature dimension from 8 to 1024 to explore its impact. From Figure 2B, we can see that the best performance is achieved when the feature dimension reaches 256. Intuitively, an appropriate feature dimension can encode enough information about the drug. When the feature dimension is too large, it can lead to overfitting of the model.

We investigated the effect of the number of channels from 1 to 3 on the model. We observe that all metrics of the model improve when the number of channels increases above 1, while they start to decrease after the number of channels reaches 3 (see Figure 2C). This indicates that an excessive number of channels causes the model to introduce too much noise information.

## 4 conclusion

In this paper, we propose a new model DDI‐Transform for DDI event prediction, which can efficiently fuse drug structure information and drug bind‐protein information to consider drug multidimensional features. And the model can use a stack of DDI‐Transform layers to adaptively learn to get new subgraphs, in which the effective association information between similar drug pairs and DDI events is fused. The experimental results show that our model DDI‐Transform outperforms SOTA DDI events prediction models.

## 5 materials and methods

### 5.1 Datasets

In order to demonstrate the effectiveness of our proposed model, we conduct extensive experiments on two real world datasets. Dataset 1 is the public dataset that Deng et al. [5] collected, containing 572 drugs with 74,528 pairwise DDIs, which are associated with 65 types of events. Dataset 2 was collected from DrugBank by Lin et al. [13] and contained 1258 drugs and 323,539 pairwise DDIs, which are associated with 100 types of events. Dataset 2 has twice as many drugs as Dataset1, four times as many DDI events as Dataset 1, and a much richer set of event types. Therefore, Dataset 2 contains more information than Dataset 1. The generalization of the proposed model can be verified using the two different scale datasets.

### 5.2 Experimental setup

The maximum number of iterations is set to 1500, and the Adam algorithm with a learning rate of 0.001 is used to optimize all training parameters by random search during each iteration. We set the number of DDI‐Transform layers to 2, the number of channels K to 3, and the node feature dimension to 256. To comprehensively evaluate our proposed model, we adopt 5‐fold cross‐validation and randomly divide the dataset into five subsets and the evaluation score is the average of the result of the five subsets. We use the early‐stopping strategy to prevent our model from overfitting by automatically stopping the model when, after 10 epochs, it does not further improve.

### 5.3 Drug structure feature extraction module

The drug structure feature extraction module uses the message passing neural network (MPNN) with attention pooling. The drug molecule can be seen as a graph structure, where each atom can be represented as a node and each bond as an edge. Consider a molecule graph represented as an undirected graph *G*_*i*_(*V*,*E*), where *V* is the set of atoms and *E* is the set of bonds. The MPNN with attention pooling has two phases: a message passing phase and a readout phase.

The message passing phase runs for *T* time steps and is defined in terms of message passing phase and message update phase. During the message passing phase, the state of each node at time step *t* is represented as $h_{v}^{t}$ and randomly initialized embedding vectors at *t* = 0. The hidden states $h_{v}^{t}$ of each node are updated using messages $m_{v}^{t+1}$ according to following equations:

(1) $$m_{v}^{t+1}=\sum_{w\in N\left(v\right)}W_{vw}^{t+1}h_{w}^{t}$$

Note that *v*, *w* are two nodes in graph *G*_*i*_; *N*(*v*) is the set of neighboring nodes of node *v*; $W_{vw}^{t+1}\in R^{N_{d}\times N_{d}}$ is a matrix of trainable parameters at the (*t* + 1)‐th step; $h_{w}^{t}$ is the hidden state of node *w*.

Inspired by [27, 28], we use two non‐linear transform functions *F* and *C*, and the following is node update function:

(2) $${\tilde{h}}_{v}^{t+1}=h_{v}^{t}⨁m_{v}^{t+1}$$

(3) $$h_{v}^{t+1}=C\left(W_{c},{\tilde{h}}_{v}^{t+1}\right)⨀h_{v}^{t}+F\left(W_{f},{\tilde{h}}_{v}^{t+1}\right)⨀m_{v}^{t+1}$$

where ⨁ denotes the concatenation operation; *W*_*c*_ and *W*_*f*_ are two learnable weight parameters; $h_{v}^{t+1}$ represents the hidden state at the (*t* + 1)‐th step for node *v*; ⨀ denotes the element‐wise product. We define $F\left(W_{f},{\tilde{h}}_{v}^{t+1}\right)$ as fuse gate and $C\left(W_{c},{\tilde{h}}_{v}^{t+1}\right)$ as carry gate because fuse gate can fuse the features of node neighbors and previous hidden state, while carry gate can carry the features of the node itself. We consider both the influence of neighboring nodes on the central node and the influence caused by the node itself in the previous step through this update process.

The message passing phase runs for *T* time steps to learn the hidden representation for every node and obtain the final hidden state $h_{v}^{T}$ for each node at the last message passing step. Then in the readout phase, the entire molecular graph is calculated as an embedding vector to represent the whole drug molecular feature. We use a simple but efficient attentive readout function to perform computations as follows:

(4) $$E_{i}^{\prime }=\sum_{v\in V}\sigma_{1}\left(W_{i1}\left(h_{v}^{0}⨁h_{v}^{T}\right)+b_{i1}\right)⨀\sigma_{2}\left(W_{i2}\left(h_{v}^{0}⨁h_{v}^{T}\right)+b_{i2}\right)$$

where ⨁ denotes the concatenation operation, *σ*_1_ and *σ*_2_ represent the sigmoid activation function and the tanh activation function respectively, $\sigma_{1}\left(W_{i1}\left(h_{v}^{0}⨁h_{v}^{T}\right)+b_{i1}\right)$ acts as a soft attention mechanism that represents the importance score of each node. *W*_*i*1_ and *W*_*i*2_ are two learnable weight parameters, and ⨀ denotes the element‐wise product.

### 5.4 Drug bind‐protein feature extraction module

**Bind‐protein feature extraction.** Drug bind‐protein features are composed of various entity features associated with the drug. Four representations of entities are used here: Transporter, Carrier, Enzyme and Target. These features bring a lot of additional information to the drug. For each feature in each drug there is a series of descriptors to represent it, so for each feature a 0 or 1 is used to represent the presence or absence of that descriptor, thus forming a binary feature vector for each feature. However, these feature vectors have high dimensionality, most of which are 0. High dimensionality input may cost too much computational resources and may induce the phenomenon of the curse of dimensionality, which can lead to extremely inferior performance for some models [29]. Based on the assumption that similar drugs may interact with each other, we use the Jaccard similarity calculated from the bit vector. The Jaccard similarity metric is then used to calculate the similarity between pairs of drugs, for which the equation is as follows:

(5) $$J\left(x_{i},x_{j}\right)=\frac{\left|x_{i}\cap x_{j}\right|}{\left|x_{i}\cup x_{j}\right|}=\frac{\left|x_{i}\cap x_{j}\right|}{\left|x_{i}\right|+\left|x_{j}\right|-\left|x_{i}\cap x_{j}\right|}$$

After obtaining the similarity matrix between drug pairs, to make the drug feature representation more dense and reduce sparsity, we conduct principal components analysis [30] to compress the features to improve the accuracy of the feature vector. We get the transporter similarity matrix $E^{t}\in R^{N_{d}\times D}$, carrier similarity matrix $E^{c}\in R^{N_{d}\times D}$, target similarity matrix $E^{a}\in R^{N_{d}\times D}$ and enzyme similarity matrix $E^{e}\in R^{N_{d}\times D}$, where *N*_*d*_ represents the number of drugs and *D* represents the dimension of the vector. Finally, we obtain four features of drug *i* through the similarity matrix $e_{i}^{t}\in E^{t}$, $e_{i}^{c}\in E^{c}$, $e_{i}^{a}\in E^{a}$ and $e_{i}^{e}\in E^{e}$, respectively.

**Auto‐encoder with self‐attention mechanism.** We then concatenate the four features into an auto‐encoder with the self‐attention mechanism. Auto‐encoder is an unsupervised neural network model, which has two parts: encoder and decoder. It can learn the hidden features from the input data. Since the dimensionality of the four features input is too high, it is necessary to reduce the dimensionality of the input features. The self‐attention mechanism is a variant of attention mechanism that relies less on external information and is better at capturing the internal correlation of features. Therefore, we add a self‐attention layer before the output layer of the encoder of the auto‐encoder. The outside‐level features embedding *E*_*i*_ of the drug *i* can then be obtained from the output of the auto‐encoder.

### 5.5 DDI‐Transform network module

Because the model needs to predict different types of interaction events, we connect drug pairs with interactions to form heterogeneous graphs, and then divide these heterogeneous graphs into subgraphs according to different types of interaction events, that is, different types of edges. In this way, we can obtain the subgraphs corresponding to each interaction event. Inspired by the principle of similarity of drug interactions and the influence of multiple DDI events, we get new subgraphs of similar drug pairs and influences between DDIs, by adaptive learning. The DDI‐Transform network module has the following steps:

**Feature similarity graph.** The drug features learned from the above two modules (via metric learning) provide the drug molecule structure similarity feature graph and the drug bind‐protein similarity feature graph. The feature similarity graph determines the probability that an edge can be formed between two nodes based on the node features, with larger values representing more similarity between the nodes. The edge between nodes is obtained by:

(6) $$S^{\mathrm{FS}}\left[i,j\right]=\left\{\begin{array}{c} \Phi^{\mathrm{FS}}\left(E_{i},E_{j}\right),\\ 0, \end{array}\right.\;\begin{array}{l} \Phi^{\mathrm{FS}}\left(E_{i},E_{j}\right)\geq \epsilon \\ \text{otherwise} \end{array}$$

where *E*_*i*_ and *E*_*j*_ correspond to the feature of drug *i* and drug *j*, respectively, and *ϵ* ∈ [0,1] is the threshold. *Φ* ^*FS*^ is a K‐head weighted cosine similarity function defined as:

(7) $$\Phi^{\mathrm{FS}}\left(E_{i},E_{j}\right)=\frac{1}{K}\sum_{k}^{K}\cos\;\left(W_{k}⨀E_{i},W_{k}⨀E_{j}\right)$$

where *W*_*k*_ is the learnable parameter matrix of *Φ* ^*FS*^.

**Meta‐path subgraph generation.** We concatenate the interaction events subgraph with two feature similarity graphs to obtain the set of subgraphs A. Inspired by meta‐path, we capture the most effective meta‐path associated between DDI events by adaptively learning to get a new meta‐path subgraph. Meta‐path denoted by *P* is a path on the heterogeneous graph *G* that is connected with heterogeneous edges, that is, $v_{1}\overset{t_{1}}{\to }v_{2}\overset{t_{2}}{\to }\text{…}\overset{t_{l}}{\to }v_{l+1}$, where *t*_*l*_ ∈ *T* ^*e*^ denotes an *l*th edge type of meta‐path. Given the composite relation or the sequence of edge types (*t*_1_, *t*_2_,..., *t*_*l*_), the adjacency matrix *A*_*P*_ of the meta‐path *P* is obtained by the multiplications of adjacency matrices as:

(8) $$A_{P}=A_{t_{1}}\text{…}A_{t_{n-1}}A_{t_{n}}$$

The meta‐path can cover multi‐hop connections and a new subgraph structure will be represented as an adjacency matrix in our framework. A new meta‐path subgraph generation requires two steps. First it needs to soft select two subgraphs $A_{t_{1}}$, $A_{t_{2}}$ from the set of adjacency matrices A. Second, it can learn a new graph structure by two relation operations (i.e., matrix multiplication of two adjacency matrices, $A_{t_{1}}A_{t_{2}}$). We need to choose two subgraphs to calculate a convex combination as $A_{1}=\sum_{t_{1}\in T^{e}}{\alpha_{t_{1}}A}_{t_{1}}$, where $\alpha_{t_{1}}$ is the weight parameter of 1 × 1 as in Figure 1 with the weights from softmax function as:

(9) $$A=\phi \left(A;\text{softmax}\left(W_{\phi }\right)\right)$$

where *ϕ* is the convolution function and $W_{\phi }\in R^{1\times 1\times K}$ is the learnable parameter of *ϕ*. The weight parameter here enables the model to adaptively select the most efficient subgraph for the prediction results and generate a new meta‐path subgraph.

The meta‐path subgraph is obtained by the DDI‐Transform layer, which is the matrix multiplication of *A*_*i*_ and *A*_*j*_. For numerical stability, the matrix is normalized by its degree matrix as *A*_*l*_ = *D* ^−1^ *A*_*i*_ *A*_*j*_. We need to learn meta‐paths of arbitrary length, which requires a stack of DDI‐Transform layers. The adjacency matrix of arbitrary length meta‐path can be calculated as follows:

(10) $$A_{l}=\left(\sum_{t_{1}\in Τ}{\alpha_{t_{1}}A}_{t_{1}}\right)\;\left(\sum_{t_{2}\in Τ}{\alpha_{t_{2}}A}_{t_{2}}\right)\text{…}\;\left(\sum_{t_{l}\in Τ}{\alpha_{t_{l}}A}_{t_{l}}\right)$$

This eventually enables the learning of a new meta‐pathway subgraph that is able to fuse similar drug pair information as well as correlation information between DDI events. To learn both long and short distance meta‐path subgraphs, we add the identity matrix to the set of subgraphs A. The identity matrix is *A*_0_ = *I*.

More than one new meta‐path subgraph is learned in the model, and there may be multiple meta‐path subgraphs that are efficient for prediction, so multiple channels are used in the model for learning, setting the number of channels *C*. Eventually the model will learn *C* new subgraphs. Finally, the GCN operation is performed on the learned subgraphs on each channel to fuse the neighbor node information of each node. The matrix concatenating on each channel is obtained to provide the final representation of each node:

(11) $$X=E^{\prime }⨁\;E$$

(12) $${\hat{E}}^{\prime }={\|}_{i=1}^{C}\sigma \left(D_{i}^{-1}A_{l_{i}}XW\right)$$

where ‖ is the concatenation operator; $A_{l_{i}}$ denotes the meta‐path subgraph *A*_*l*_ on the *i*th channel; *D*_*i*_ is the degree matrix of $A_{l_{i}}$; *X* is the feature matrix of drug structure features and drug bind‐protein features concatenated; *W* is a trainable weight matrix. ${\hat{E}}^{\prime }$ contains the node features on *C* different meta‐path subgraphs.

### 5.6 Interaction prediction

The features of two drugs are extracted in ${\hat{E}}^{\prime }$. Then the two drug features are concatenated and fed into a MLP consisting of a two‐layer fully connected neural network for learning:

(13) $${\hat{E}}_{ij}^{\prime }={\hat{E}}_{i}^{\prime }\;⨁\;{\hat{E}}_{j}^{\prime }$$

(14) $${\hat{y}}_{ij}=\sigma \left(W_{4}\mathrm{ReLU}\left(W_{3}{\hat{E}}_{ij}^{\prime }+b_{3}\right)+b_{4}\right)$$

where *W*_3_ and *W*_4_ are trainable parameters; *b*_3_ and *b*_4_ are bias vectors; *σ* is the softmax activation function; ${\hat{y}}_{ij}$ is the final predicted value. Cross‐entropy is used as the loss function for training.

## AUTHOR CONTRIBUTIONS

**Jiaming Su**: Data curation; methodology; visualization; software; writing – original draft. **Ying Qian**: Conceptualization; supervision; writing – review and editing.

## CONFLICT OF INTEREST STATEMENT

The authors Jiaming Su and Ying Qian declare that they have no conflicts of interest.

## ETHICS STATEMENT

This article does not contain any studies with human or animal subjects performed by any of the authors.

## Data Availability

The data are open source and available from the corresponding article that mentioned.
